# Cancer after cholecystectomy: record-linkage cohort study

**DOI:** 10.1038/sj.bjc.6602392

**Published:** 2005-03-15

**Authors:** M J Goldacre, J D Abisgold, V Seagroatt, D Yeates

**Affiliations:** 1 Unit of Health-Care Epidemiology, Department of Public Health, University of Oxford Old Road Campus, Old Road, Oxford OX3 7LF, UK

**Keywords:** cholecystectomy, colon cancer, pancreatic cancer, stomach cancer, liver cancer, record linkage

## Abstract

We investigated whether cholecystectomy is associated with subsequent cancer and, if so, whether the association is likely to be causal, by undertaking a retrospective cohort study using linked medical statistics, comprising a cholecystectomy group (*n*=39 254) and a reference cohort admitted for a range of other medical and surgical conditions (*n*=334 813). We found a short-term significant elevation of rates of cancers of the colon, pancreas, liver, and stomach after cholecystectomy, but no long-term elevation. Excluding colon cancers within 2 years of admission to hospital, the rate ratio for colon cancer after cholecystecomy, compared with the reference cohort, was 1.01 (95% confidence interval 0.90–1.12) and after 10 years or more follow-up it was 0.94 (0.79–1.10). It is highly improbable that the short-term associations between cholecystectomy and gastrointestinal cancers are causal, and we conclude that cholecystectomy does not cause cancer.

The geography of high prevalence of gallstones is thought to reflect, in part, the effects of a western lifestyle and diet (Burkitt, 1970, 1975), raising the possibility that some cancers in patients with gallstones are attributable, at least in part, to lifestyle-related aetiology. In addition, certain autopsy and case–control studies have suggested that cholecystectomy may cause colon cancer, perhaps as a result of alteration of bile flow (Coleman, 1991; Giovannucci *et al*, 1993; Reid *et al*, 1996). However, cohort studies, which are less prone to biases than the other two designs, have been less commonly undertaken.

In some of the positive case–control studies, the time scale between cholecystectomy and colon cancer was either unreported or fairly short. If cholecystectomy is a cause of cancer, an increase in cancer is likely to be most apparent at long time intervals after operation. We have undertaken a record-linkage cohort study of cancer in people after cholecystectomy. We aimed to determine whether there are distinctive patterns of cancer in people who have undergone cholecystectomy; and, for any cancers with an elevated rate after cholecystectomy, address the question of causality.

## MATERIALS AND METHODS

We used data from the Oxford record-linkage study (ORLS), which includes brief statistical abstracts of records of all hospital admissions (including day cases) in National Health Service (NHS) hospitals, and of all deaths regardless of where they occurred, for defined populations within the former Oxford NHS region from 1 January 1963 to 31 March 1999. The hospital data were collected routinely in the NHS as the Region's hospital discharge statistics, while the mortality data derive from death certificates. Data collection covered part of one health district and its associated hospitals from 1963 (population 350 000), two districts from 1966 (population 850 000), six districts from 1974 (population 1.9 million) and all eight districts of the region and their associated hospitals from 1983 (population 2.5 million). With the approval of the Oxford Region's Data Protection Steering Group, the data for each individual were linked together routinely, on an ongoing basis, as part of the region's health information system. They are now anonymised and archived. The cholecystectomy cohort was obtained by selecting records of individuals aged 15–84 years who underwent this operation. A reference cohort, to compare with the cholecystectomy cohort, was constructed by similarly selecting records of individuals aged 15–84 years admitted for a wide range of other medical and surgical conditions. This is our ‘reference group’ of patients that has been used in other studies of inter-relationships between diseases (Goldacre *et al*, 2000). We considered that the incidence of cancer in the reference cohort would approximate to that in the general population of the region while allowing for migration from it (data on migration of individuals were not available). We excluded all people with a cancer recorded at the same admission as that for cholecystectomy or reference cohort conditions. We then searched the database for any subsequent record of cancer in the cholecystectomy cohort and the reference cohort.

### Statistical methods

For each cancer site, in comparing the rate of subsequent cancers in the cholecystectomy and reference cohorts, we took ‘date of entry’ into each cohort as date of first admission for cholecystectomy or reference condition, and the ‘date of exit’ for each individual patient as the date of subsequent admission for the cancer, or death, or 31 March 1999, whichever was the earliest. For each specific cancer, in comparing the cholecystectomy and reference cohorts, we first calculated cancer incidence rates, standardised by age (in 5-year age groups), sex, calendar year of first recorded admission, and district of residence, taking the combined cholecystectomy and reference cohorts as the standard population. This standardisation was undertaken to ensure that the populations under comparison were equivalent in these respects. We then calculated the ratio of the standardised rate of occurrence of the cancer in the cholecystectomy cohort relative to that in the reference cohort. The confidence interval for the rate ratio and statistics for its significance were calculated as described elsewhere (Breslow and Day, 1987).

People in each 5-year age group who underwent cholecystectomy were compared with as many people with the reference conditions as there were in the ORLS data set in each age group, in order to maximise statistical power and minimise the confidence intervals around the risk ratios. We divided time intervals from admission for cholecystectomy to admission for cancer into less than 1 year, 1–2 years, 2–4, 5–9, and 10 years or more. In most of the results presented, we excluded cancers that occurred within 2 years of the admission for cholecystectomy to minimise the inclusion of cancers that were probably coincidental to rather than caused by cholecystectomy.

## RESULTS

There were 39 254 individuals in the cholecystectomy cohort and 334 813 in the reference cohort. The rate ratio for colon cancer after cholecystectomy, compared with the reference cohort, was 1.12 (95% confidence interval 0.99–1.24) overall. Excluding cases of colon cancer diagnosed in the first year after cholecystectomy, the rate ratio was 1.04 (0.92–1.15). Excluding cases diagnosed in the first 2 years after operation, the rate ratio was 1.01 (0.90–1.12; Table 1). For those cases with at least 10 years follow-up, the rate ratio was 0.94 (0.79–1.10) based on 147 observed and 156.4 expected cases.

Overall, the rate ratio after cholecystectomy was significantly high for cancer of the pancreas (rate ratio 1.88; 95% confidence intervals 1.56–2.12), liver (1.45; 1.09–1.90), and stomach (1.23; 1.06–1.42). Following exclusion of cancers within 2 years of cholecystectomy, no cancer showed a significantly high rate ratio (Table 1). There was a small but significant deficit of breast cancer after operation in the data overall (0.87; 0.79–0.96) and when cases of breast cancer within 2 years of cholecystectomy were excluded (0.89; 0.80–0.97; Table 1).

## DISCUSSION

Since gallstones are common in western populations, they will often be found by chance in people who present with abdominal pain caused by other factors such as cancer. It is well established that early manifestations of abdominal cancer are sometimes misdiagnosed as gallstones and treated with cholecystectomy. For example, recent studies have shown that, of patients readmitted within a few months of laparoscopic cholecystectomy, a common cause of readmission is colon cancer (Gal *et al*, 1998; Malouf *et al*, 2000; Wysocki *et al*, 2001).

The literature on whether there is a causal association between cholecystectomy and colon cancer is inconsistent. It is clear, however, that the most markedly positive association has come from the studies with designs that are most susceptible to bias. The choice of controls may influence the results of case–control studies and cohort studies with long-term follow-up are more satisfactory. In 1993, Giovannucci *et al* (1993) published a meta-analysis of 33 case-cohort and 6 cohort studies. The pooled results from the 33 case–control studies showed a significant positive association between cholecystectomy and colorectal cancer (relative risk (RR) 1.34, 95% confidence interval 1.14–1.57). If, however, there are systematic biases in such studies that tended to go in the same direction, pooling the studies will simply combine the biased findings. The meta-analysis found no significant association between cholecystectomy and colon cancer in the combined results from the six cohort studies (RR 0.97, 0.82–1.14; Giovannucci *et al*, 1993). Another meta-analysis has since reported very similar findings (Reid *et al*, 1996), while three more large cohort studies with long follow-up have also reported on the subject. A study from Sweden (Lagergren *et al*, 2001) updating previous Swedish studies (Adami *et al*, 1987; Ekbom *et al*, 1993) reported a significant but small increase in risk of intestinal cancer after cholecystectomy. A study from Holland with relatively few cancers reported an increased risk of colorectal cancer (Goldbohm *et al*, 1993). A US study of 85 184 women followed for 16 years reported a non-significant increase in the risk of colon cancer overall after cholecystectomy, with the highest risk in the proximal colon at the shortest reported time (of less than 5 years) interval after the operation (Schernhammer *et al*, 2003). This study also reported a significant increase in cancer of the rectum. The results of these three as well as the previous cohort studies have not removed uncertainty about any causal association between cholecystectomy and colon cancer. Other cancers, too, have been studied after cholecystectomy, but the evidence on causality associations with cancers of the pancreas, liver, and stomach is also inconsistent (Ekbom *et al*, 1996; Johansen *et al*, 1996; Chow *et al*, 1999; Schernhammer *et al*, 2002).

The strengths of our study are that it is a large cohort study; records of cholecystectomy and of cancer were created independently and only subsequently brought together; it examines different time intervals from operation to cancer, with many cases of cancer at long time intervals after operation; and it was undertaken in a geographically defined, but otherwise unselected, population. Most forms of bias that can occur in case–control studies – such as selection, referral, or responder bias – are unlikely or impossible in a population-based cohort study.

The study has some weaknesses. We have no information about the criteria used for cancer diagnosis, or about when, prior to admission for cancer, this diagnosis was first suspected or made. The database of hospital records is confined to hospital inpatients or to day cases, which we believe would identify all cases of cholecystectomy, and the great majority of cancer cases would be identified by these sources. We do not have records of admission or death in people who migrated out of the area after cholecystectomy or after admission for the reference conditions. We assumed that outward migration is not appreciably different in the cholecystectomy and reference cohorts. The fact that the rate ratios shown in the Table 1 are generally close to one, both for those cancers where there was a prior hypothesis about an association and for those cancers outside the gastrointestinal tract where there was not, suggests that the reference cohort is not materially biased with respect to migration. If it were, cancer rates in general would have been systematically high or low. It also suggests, more generally, that the reference cohort is an appropriate comparator with respect to its composition. We consider that the slight deficit of breast cancer in association with cholecystectomy, although statistically significant, is probably due to chance.

We found significantly elevated rates of several intra-abdominal cancers overall but, after excluding cancers within 2 years of cholecystectomy, there was no elevation of rates, making it very unlikely that these associations are causal. The rate ratios of cancers in the cholecystectomy cohort, compared with the reference cohort, did not increase with time from operation. Our findings add to the evidence that cholecystectomy probably does not cause cancer.

## Figures and Tables

**Table 1:** Cancers in people who underwent cholecystectomy, excluding cancers in the first 2 years after admission for cholecystectomy or reference condition^a^

**Cancer site or type (ICD code^b^)**	**No. in reference cohort with each cancer**	**No. observed in cholecystectomy cohort**	**No. expected in cholecystectomy cohort**	**RR (95% CI)**
Oesophagus (150)	803	91	93.2	0.98 (0.79–1.21)
Stomach (151)	1354	177	159	1.11 (0.96–1.29)
Colon (153)	2324	320	318	1.01 (0.90–1.12)
Rectum (154)	1407	185	178	1.04 (0.89–1.20)
Liver (155)	306	38	41.7	0.91 (0.64–1.25)
Pancreas (157)	791	127	120	1.06 (0.88–1.26)
Lung (162)	4222	447	480	0.93 (0.85–1.03)
Malignant melanoma (172)	337	57	48.6	1.17 (0.39–1.52)
Other skin (173)	1777	225	224	1.00 (0.86–1.12)
Breast (174, 175)	2285	434	486	0.89 (0.80–0.97)
Cervix (180)	205	57	46.1	1.24 (0.94–1.60)
Uterus (182)	379	94	83.5	1.13 (0.91–1.38)
Ovary (183)	126	88	92.5	0.95 (0.76–1.17)
Prostate (185)	2193	215	195	1.10 (0.94–1.23)
Kidney (189.0, 189.1)	457	60	53.1	1.13 (0.86–1.45)
Bladder (188)	1641	168	174	0.97 (0.83–1.12)
Brain, malignant (191)	392	51	47.7	1.07 (0.80–1.41)
Lymphoma (200–202)	825	98	98.7	0.99 (0.81–1.21)
Multiple myeloma (203)	444	64	56.7	1.13 (0.87–1.44)
Leukaemia (204–208)	680	96	85.5	1.12 (0.91–1.37)
				
All cancers (140–208)	22 703	2921	2966	0.98 (0.95–1.02)

a Number of people with each cancer in the reference cohort and in the cholecystectomy cohort, number expected in the cholecystectomy cohort, ratio of rates (RR) in the cholecystectomy cohort compared with the reference cohort, and 95% confidence intervals (CI) on the ratio of rates.

b International Classification of diseases, 9th Revision, and equivalent codes in Revisions 7, 8, and 10.

The table includes all individual cancers for which there were at least 30 expected or 30 observed cases. Of the cancers not tabulated, none showed a significant association with cholecystectomy. Results are available on request from the authors.
